# Phthalate Esters in Tap Water, Southern Thailand: Daily Exposure and Cumulative Health Risk in Infants, Lactating Mothers, Pregnant and Nonpregnant Women

**DOI:** 10.3390/ijerph19042187

**Published:** 2022-02-15

**Authors:** Kingsley Ezechukwu Okpara, Khamphe Phoungthong, Iwekumo Agbozu, Edeh Edwin-Isotu, Kuaanan Techato

**Affiliations:** 1 Environmental Assessment and Technology for Hazardous Waste Management Research Center, Faculty of Environmental Management, Prince of Songkla University, Songkhla 90112, Thailand; okparakingsley777@gmail.com (K.E.O.); kuaanan.t@psu.ac.th (K.T.); 2 Department of Chemistry, University of Africa, Toru-Orua 561101, Nigeria; iwekumo.agbozu@uat.edu.ng; 3 Centre for Environmental Management and Control, University of Nigeria, Enugu 400001, Nigeria; edisotu@gmail.com

**Keywords:** phthalate esters, raw and tap water, fate of PAEs, susceptible groups and cumulative risk assessment

## Abstract

Human exposure to phthalate esters (PAEs) via drinking water has generated public health concerns due to their endocrine disruptive abilities. This study reports on the occurrence and fate of six PAEs in raw and tap water samples collected from provincial waterworks located in Songkhla Province, Southern Thailand. In addition, the daily exposure and cumulative health risk of susceptible populations due to drinking tap water were evaluated by using four different reference dose (RfDs) sources. The maximum concentrations of PAEs in raw water were between 1.68 and 4.84 and 0.52 and 1.24 µg/L in tap water. Moreover, the levels of PAEs in the tap water samples indicated the poor PAEs removal efficiency of the conventional treatment process (59.9–69.1%). The contribution of water to the daily intake of PAEs did not exceed 0.37% in all the groups. Furthermore, both the individual and cumulative risk assessment showed negligible noncarcinogenic and antiandrogenic risk for all the groups. Nevertheless, the cumulative risk showed an increasing trend in the order of infants > lactating mothers > pregnant women > nonpregnant women, suggesting that infants are more vulnerable. In additional, the newly proposed RfDAA yielded higher hazard quotient and hazard index estimates, which indicates it is a more sensitive tool than other RfDs for the assessment of the individual and mixture risk of pollutants. The carcinogenic risk of DEHP was acceptable in every group. However, we recommend a future cumulative risk assessment of vulnerable groups considering their simultaneous exposure to all chemicals that have antiandrogenic effects via tap water.

## 1. Introduction

Phthalate esters (PAEs) are synthetic compounds that have evoked interest in recent decades due to their ubiquitous environmental contamination, endocrine-disrupting effects, and potential adverse effects on public health. PAEs are mainly used as plasticizers in the manufacturing of plastics, rubber, polyvinyl chloride (PVC), and other polyethylene products to improve their flexibility, workability, and durability [1,2,3]. PAEs are endocrine-disrupting chemicals (EDCs), whose effects may not appear until long-term exposure [4,5,6]. Prolonged exposure to PAEs, especially through drinking water, may result in adverse health conditions, including endocrine system disruption, cancer, developmental abnormalities, and polyneuropathy [7,8,9,10]. In additional, chronic consumption of PAE-contaminated water has been associated with endocrine-disrupting activity [6,9,10,11]. Moreover, to reduce the public health risk of PAE contamination in drinking water, some international organizations and countries have established regulations and standards limits for some species of PAEs in drinking water. The United State Environmental Protection Agency (USEPA) and World Health Organization (WHO) recommend a maximum contaminant level (MCL) value of 6 and 8 μg/L for DEHP, respectively. In China, the MCLs of some congeners of PAEs in drinking water have been stipulated including those of DBP (3 µg/L), DEHP (8 µg/L), and DEP (300 µg/L) [3,12].

Exposure to PAEs via drinking tap water from public water distribution systems such as waterworks is an emerging area of public health concern. Studies have reported elevated PAEs in surface waters serving as municipal source waters for provincial waterworks [12,13,14,15]. In addition, PAEs in source waters are the main contributors to drinking water [10,14]. However, PAEs may also leach into public water distribution systems built with plastic high-density polyethylene (HDPE) or polyvinyl chloride (PVC) pipes [16]. Moreover, the occurrence of PAEs in tap water has been attributed to the inadequate removal of PAEs by conventional drinking water treatment plants, leading to a potential source of human exposure to PAEs [13,15]. Furthermore, previous studies observed that tap water ingestion and absorption are the major sources of human exposure to PAEs [16,17,18,19]. Nevertheless, PAEs whose adverse effects on public health have been established are not readily included in the common drinking water standards released by regulatory bodies, especially in developing countries.

In developing countries, most of the municipal drinking water treatment plants use conventional treatment processes, including coagulation, sedimentation filtration, and chlorination [6,10,15,20]. These treatments cannot altogether remove PAE contaminants in raw water. Therefore, PAEs may persist in tap water after treatment. In addition, the occurrence of PAEs has been reported in water bodies that supply raw waters to provincial waterworks in developing countries [12,13,15,21,22,23,24,25]. In addition, while there is a growing tendency toward replacing tap water with bottled water in developed countries, the reverse is the case in developing countries. Notably, the regulations or standards for surface and drinking water in most developing countries do not specify PAEs [13,23]. The lack of rules and standard limits of drinking water may impede the eradication of human exposure to PAEs via drinking water. Information on the levels and fate of PAEs, as well as human exposure to them, particularly the exposure of vulnerable groups via drinking tap water, is critical to the formulation of policies related to public health challenges. In addition, robust scientific data on exposure and risk assessments of pollutants are used by risk managers to determine the need for regulation or remediation and to set discharge limits.

The exposure of vulnerable subgroups to PAEs is generating serious public health concern. Pregnancy and lactation periods across the lifespan have been recognized as potentially critical windows of vulnerability to exposure to a variety of chemicals [2,26,27,28]. Maternal exposure to PAEs during pregnancy has been associated with alterations in hormones that play key roles in pregnancy maintenance and fetal growth and development, such as testosterone, progesterone, and thyroid hormone [29,30,31,32]. Additionally, the exposure of fetuses and infants is of primary concern since this group is extremely sensitive to the effects caused by chemicals with hormone-like properties including PAEs [33,34]. Epidemiological studies revealed that PAEs may cause varying adverse effects in humans, particularly the sensitive groups [35,36,37,38,39]. A major chronic exposure pathway of PAEs to the human body is via drinking water, which includes tap water.

Considering the potential risk of PAEs, it is essential to study the risks associated with exposure to PAEs and have a better understanding of the key sources of exposure to these compounds. Very few studies have been conducted to evaluate the level and fate of PAEs in municipal waterworks and the daily intake of PAEs by humans who drink tap water. Furthermore, there have been no studies regarding the associated potential risks (carcinogenic or noncarcinogenic) from tap water consumption focusing on vulnerable groups including infants, lactating mothers, and pregnant women, despite their high sensitivity to the adverse effects of PAEs. In order to find a suitable answer as to whether tap water produced by waterworks that use a conventional water treatment process jeopardizes the health of its consumers, the present study aims to (1) evaluate the concentration and composition of PAEs in the raw and tap water from waterworks, (2) examine the removal efficiencies of PAEs by waterworks that use a conventional treatment process; (3) assess the exposure and cumulative health risk of PAEs in susceptible groups via ingestion of tap water, and (4) determine the contribution of tap water to the daily intake of PAEs.

## 2. Materials and Methods

### 2.1. Sample Collection

**Figure 1:**
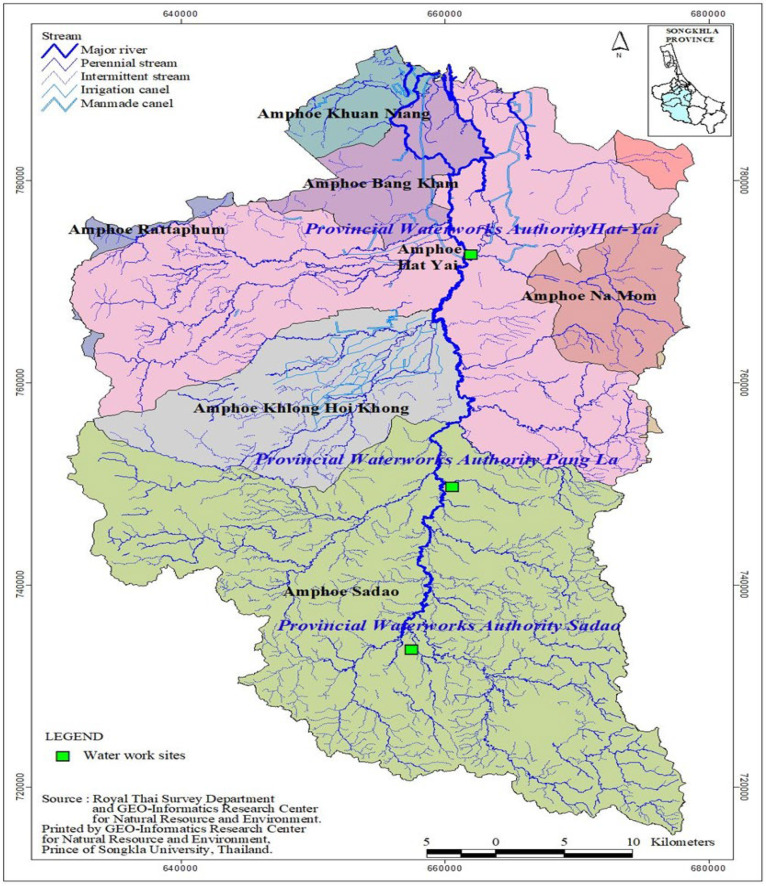
Map showing the three provincial waterworks location along U-Tapao River in Songkhla Province.

Raw and tap water samples were drawn from provincial waterworks in the study areas. The provincial waterworks are shown in Figure 1. Raw water was collected at 0.5 m below each site of the raw water wells, representing the mixed water columns. Tap water samples were taken from the distribution points of each waterwork. Water samples were collected in 1-liter pretreated bottles in triplicate, placed in an icebox, transferred to the laboratory, and stored in 4 °C refrigerators in the laboratory until analysis. An aliquot of 120 µL of 0.75 g/mL sodium thiosulfate solution was placed in 1 L of tap water to block the chlorine content of tap water and prevent it from forming interference, which would affect the analysis. All samples were extracted within two days and analyzed within three days.

### 2.2. Chemicals and Reagents

PAE standards used in this present study include di-n-butyl phthalate (DBP), benzyl butyl phthalate (BBP), di-2-ethyl hexyl phthalate (DEHP), di-n-octyl Phthalate (DnOP), di-isononyl phthalate (DiNP), and diisodecyl phthalate (DIDP) and were purchased from AccuStandard Inc. (New Haven, CT, USA). All solvents, including hexane, methanol, acetone, and dichloromethane, were high-performance liquid chromatography (HPLC) grades purchased from Waters Cooperation, USA. Additionally, solid-phase extraction cartridge Florisil (1 g 6cc, Chrom, and Sep) and an internal standard of 100 mg/L benzyl benzoate (BBZ) in n-hexane were purchased from Dr. Ehrenstorfer Gmbh (Augsburg, Germany). Anhydrous sodium sulfate (Tianjin Chengguang Chemical Reagent Co., Tianjin, China) was cleaned at 600 °C for 6 h and then kept in a desiccator before use.

### 2.3. Sample Pretreatment

The pretreatment procedures for water samples were described as defined by [15,23,40]. Here, we briefly present these procedures. Before solid-phase extraction, each sample was spiked with the surrogate standards, and 1 L of each water sample was filtered via glass fiber filters (GF/F, 0.7-μmpore size, Whatman plc.). Florisil cartridges (1 g 6cc, Chrom, and Sep) were used to extract the six targeted PAE congeners from the water samples. Finally, the extracts were reconstituted with l mL of n-hexane, and benzyl benzoate was added as an internal standard before the GC–MS analysis.

### 2.4. Instrumental Analysis

The extracted PAE congeners were analyzed using gas chromatography fixed to a mass spectrometer (GC-MS), Agilent model 6890N GC–5973 MSD (Agilent Technologies, Inc. Santa Clara, CA, USA). Extracted samples were injected into the GC equipped with an HP-5 MS fused silica capillary column (30 m × 0.25 mm × 0.25 μm film thickness) and an Agilent 5973 MS detector, operating in the selective ion monitoring mode. The column temperature was initially set at 80 °C for 1 min, then ramped at 15 °C for 1 min to 300 °C and held constant for 10 min. The transfer line and the ion source temperature were maintained at 280 and 250 °C, respectively. Helium was used as the carrier gas at a flow rate of 1 mL/min. Automated samplers injected the liquid extracts of 1.0 μL in splitless mode with a venting time of 1.15 min with an inlet temperature of 300 °C. The concentrations in the water were normalized to a dry-weight basis.

### 2.5. Quality Control/Assurance

To avoid PAE contamination risks, all glassware was washed and rinsed in ethanol and subsequently heated at 350 °C for eight hours. Low PAEs—those containing reagents such as n-hexane, ethyl acetate, and methanol—were used in the present study. The quick evaporation process decreased system blanks with n-hexane, ethyl acetate, and methanol; the two reagents have relatively low evaporation points. Procedural blanks and spiked samples were processed along with each extraction round of 5 samples. The limit of detection (LOD) and limit of quantification (LOQ) for individual PAE congeners were estimated based on a signal-to-noise ratio of 3 and 10 times, respectively. All relative standard deviation (RSD%) for PAEs analyzed in water was less than 15%, indicating that all values evaluated are within the acceptance criteria. The quality assurance and quality control parameters are shown in Table 1. Besides, the chromatographs are shown in Figures S1–S4.

### 2.6. PAE Removal Efficiency by Conventional Treatment Process

One of the major steps in reducing human exposure and potential health risk of endocrine-disrupting chemicals such as PAEs is the efficient removal or elimination of PAEs in water [10]. In this study, the PAE removal efficiency of the conventional treatment process was calculated by using Equation (1):

C_(tap water) = C_(raw water × (100% − RE))(1)

where C_(tap water) represents concentration of PAEs in tap water, C_(raw water) is the concentration of PAEs in raw water, and RE is PAE removal efficiency of the conventional water treatment process.

### 2.7. Health Risk Assessment

In order to confirm that tap water collected from the three provincial waterworks in Songkla Province is safe for consumption from the viewpoint of carcinogenic and non-carcinogenic effects, daily intakes of detected PAEs including DBP, DEHP, and DiNP were estimated based on their maximum concentrations. In addition, the risk characterization was determined based on the maximum values for carcinogenic and noncarcinogenic effects in vulnerable groups (infants, pregnant women, and lactating mothers) and nonpregnant women. The risks posed by the studied compounds were estimated based on toxicity data obtained from the Integrated Risk Information System (IRIS) of the United States Environmental Protection Agency (USEPA) and the World Health Organization (WHO). The health risk was calculated based on the volume of water consumed, on average, by members of the target groups. Four different reference dose sources were used in this study to estimate the health risk. These include tolerable daily intake (TDI) values estimated by the European Food Safety Authority (EFSA), the reference doses (RfD) estimated by the USEPA, and anti-androgenicity reference doses (RfDAA) and new antiandrogenicity reference doses (NRfDAA) as estimated by [41,42]. The values of each reference dose (RfD) per congener of PAEs used for the estimation of health risk in this study including the body weight and volume of water consumed by the vulnerable groups, are indicated in Tables S1 and S2. The estimates of daily exposure to PAEs via ingestion of tap water were calculated for the target groups by applying the equation below:

EDI = (MC × WIR)/BW(2)

where EDI is the estimated daily intake of PAEs via ingestion of tap water (μg/kg body weight/day) and provided in units of liters per day; MC (μg/L) stands for the maximum concentration of DBP, DEHP, and DiNP in the tap water taken from the distribution points of the investigated waterworks; water intake rate, abbreviated as WIR, is the required volume of daily drinking water for the target group members; and BW is the body weight (kg).

In this study, water consumption rates and the values for body weight (kg) for infants, pregnant women, lactating mothers, and nonpregnant women were taken from the EPA’s Exposure Factors Handbook (EFH) (2011) and the Panel on Dietary Reference Intakes for Electrolytes and Water by Institute of Medicine (Washington, DC, USA) [43,44]. These values are indicated in Supplementary Table S1. Noncarcinogenic and antiandrogenic health risks were assessed using the hazard quotient (HQ) equations and four different RfDs as shown below [45]:

HQ = EDI/RfD = EDI/RfDAA = EDI/NRfDAA = EDI/TDI(3)

where HQ represents the hazard quotient. EDI is the estimated daily intake via drinking tap water (μg/kg body weight/day). The four reference doses used in the estimation of the HQs include EFSA TDI, USEPARfD, RfDAA, NRfDAA (μg/kg body weight/day). HQ values less than 1 indicate acceptable risk for the particular considered endpoint, whereas HQ values higher than 1 suggest unacceptable health risk.

HIRfD = HQDBPRfD + HQDEHPRfD + HQDiNPRFD(4)

HIRfDAA = HQDBPRfDAA + HQDEHPRfDAA + HQDiNPRfDAA(5)

HINRfDAA = HQDBPNRfDAA + HQDEHPNRfDAA + HQDiNPNRfDAA(6)

HITDI = HQDBPTDI + HQDEHPTDI + HQDiNPTDI(7)

The four hazard index (HI) equations above were used for the estimation of the cumulative risk resulting from the exposure to DBP, DEHP, and DiNP of infants, lactating mothers, and pregnant and nonpregnant women. HI is a regulatory method used to conduct the cumulative risk assessment (CRA) of chemicals based on the dose addition concept. It can be defined as the summation of HQs for individual chemicals with the same endpoint [41]. For this reason, the TDI, RfDs, RfDAA, and New RfDAA were used to evaluate the effects of combined exposures to PAEs in each of the provincial waterworks. The HI value was used to estimate the noncarcinogenic or antiandrogenic risk. The higher the HI value is, the greater the hazard indicated. An HI value below 0.1 indicates noncarcinogenic human exposure risk. However, an HI value between 0.1 and 1 indicates a relatively low noncarcinogenic health risk. An HI value higher than 1 indicates unacceptable health risk [46]. 

The contribution to the daily intake of these compounds via consumption of drinking water was calculated based on the following formula:

CVD = EDI/TDI × 100(8)

where CVDs represent contribution of PAEs via drinking tap water. EDI is the estimated daily intake via drinking tap water (μg/kg body weight/day). EFSA TDI is the tolerable daily intake (μg/kg body weight/day).

The cancerogenic health risk of DEHP via drinking tap water was also calculated by applying Equation (9).

Excess Cancer Risk (ECR) = CSF × EDI(9)

where Excess Cancer Risk is associated with the excess level of risk of developing cancer by being exposed to particular chemicals via specified routes; EDI is the estimated daily intake via drinking tap water (μg/kg body weight/day). CSF stands for cancer slope factors and is used to estimate the risk of cancer associated with the oral exposure to either a carcinogenic or a potentially carcinogenic substance. The CSF for DEHP is 1.4 × 10^−2^ per mg/kg·day. Generally, a cancer risk value ranging between 10^−5^ and 10^−6^ is deemed to be acceptable [47].

### 2.8. Statistical Analysis

SPSS (20.0 version, IBM, Chicago, IL, USA) was used to perform the descriptive statistic, and Spearman’s correlation coefficient was used to examine the relationship between the individual congeners and total PAEs detected in raw water.

## 3. Results

### 3.1. Occurrence and Fate of PAEs in Treatment Plants

The provincial waterworks investigated in this study include Sadao (SA), Phang La (PL), and Hat Yai (HY). The concentrations of PAEs in raw water samples used in the production of tap water are presented in Table 2. Three congeners of the target PAEs including DBP, DEHP, and DiNP were identified and quantified in the raw water, while BBP, DnOP, and DIDP were not detected. The total PAE concentrations in raw water ranged from 1.84 to 6.46, 1.69 to 5.64, and 2.88 to 10.67 µg/L for SA, PL, and HY, respectively. In addition, the maximum concentration (MC) of individual PAEs in raw water are indicated in Table 2. The MC levels of DBP were 2.04, 1.82, and 3.36 µg/L for SA, PL, and HY, respectively. For DEHP, the MC values were SA (2.68), PL (2.14), and HY (4.48 µg/L), while the values of DiNP were 1.74 (SA), 1.68 (PL), and 2.47 µg/L (HY). In this present study, the maximum concentrations of DEHP (4.48 µg/L) and DBP (3.36 µg/L) in raw water samples were higher than standard values stipulated by USEPA (PAEs, 3.0 µg/L) and WHO (DEHP, 1.3 µg/L) for surface water quality values. In addition, the DBP level was slightly higher than the stipulated Chinese surface water quality level for DBP (3 µg/L).

The correlations between DEHP, DBP, and DiNP concentrations with total PAE levels in raw water samples are shown in Table S3. The results reveal that significant correlations existed between DEHP, and DiNP, and Σ3PAE (correlation coefficients r = 0.869 and 0.804, *p* < 0.01, respectively). The correlation coefficient revealed the importance of DEHP in the total concentrations of PAEs and can be used as a marker to predict the concentration of other PAE congeners in the investigated source water.

The three detectable PAE relative contributions or profile patterns in raw water samples for DBP, DEHP, and DiNP in SA, PL, and HY, are presented in Figure S5A–C. It is clear that DEHP was most abundant in the raw water, with contributions of 41.5, 37.9, and 45.4% to total PAE loads in the water for SA, PL, and HY, respectively, followed by DBP and DiNP accounting for 31.6, 32.3, and 31.5% and 26.9, 29.8, and 23.5%. As shown in Table S5, the most crucial congener in the tap water was DEHP, with a relative composition of 40.8, 41.5, and 37.6% for SA, PL, and HY, respectively, suggesting the highest relative composition of total PAE concentrations in the tap water, followed by DBP with 32.5, 33.2, and 32.4% for SA, PL, and HY and DiNP compositions of 30 for HY, 26.7 for SA and 25.3% for PL.

A total of three PAEs were identified and quantified in the treated water samples collected from the storage tank of the three waterworks, including DBP, DEHP, and DiNP. The other PAEs (BBP, DnOP, and DIDP) were of minor significance, and their levels were all below the limit of detection. As shown in Table S4, the removal rate of PAEs by the three waterworks ranged from 59.9% to 69.1%, which varied without stable removal efficiencies. The highest removal efficiency was reported for DiNP (69.1%) in PL. The lowest removal efficiency was observed for DBP in the HY, at 59.9%. This result agrees with previous studies that reported the low removal efficiency of these groups of PAEs by conventional techniques [13,15,48].

As indicated in Table S5, the total PAE concentrations in tap water ranged from 0.59 to 2.40, 0.53 to 1.99, and 1.02 to 3.30 µg/L for Sadao, Phang La, and Hat Yai waterworks, respectively. The maximum concentration values of individual PAEs detected in tap water for DEHP were 0.98 (SA), 0.85 (PL), and 1.24 µg/L (HY). For DBP the MC levels were SA (0.78), PL (0.68), and HY (1.07 µg/L), and DiNP levels were 0.64 (SA), 0.52 (PL), and 0.99 (HY). The total levels of PAEs in tap water in this study were higher than those reported for France (0.427 μg/L) [18] and comparable with those reported for Spain (1.034 µg/L) [49]. In contrast, [11] observed much higher levels of PAEs in tap water in China than the values reported in this work. The measured concentrations of PAEs in tap water samples collected from the waterworks showed a slight variation. DEHP, DBP, and DiNP concentrations in the HY were higher than the levels in the SA and PL. This may be attributed to the location of the HY waterworks in a densely populated urban area.

### 3.2. Comparison with Studies in Other Locations

Several studies have reported the concentrations of DBP and DEHP in raw and tap water; however, the data on DiNP are scarce. In this study, the results of DBP and DEHP levels in raw and tap water obtained from conventional and advanced water treatment plants published in the literature are presented in Table 3. A comparison of available data, as shown in Table 3, revealed that the concentration of DEHP and DBP in raw and tap water samples in this present study were higher than those reported for East China and Taiwan [6,15] and lower than the levels reported for South Carolina, United States of America, and Harbin city, Northeast China [13,50]. The removal efficiencies of studied PAEs in this present study are comparable with previous studies that used a conventional treatment process. As indicated in Table 4, the available data show that the concentrations of DEHP and DBP in tap water samples that had undergone conventional treatment processes were much higher than those reported for tap water in advanced treatment processes, indicating a more effective treatment or removal of PAEs.

### 3.3. Estimated Daily Intake and Risk

**Figure 2:**
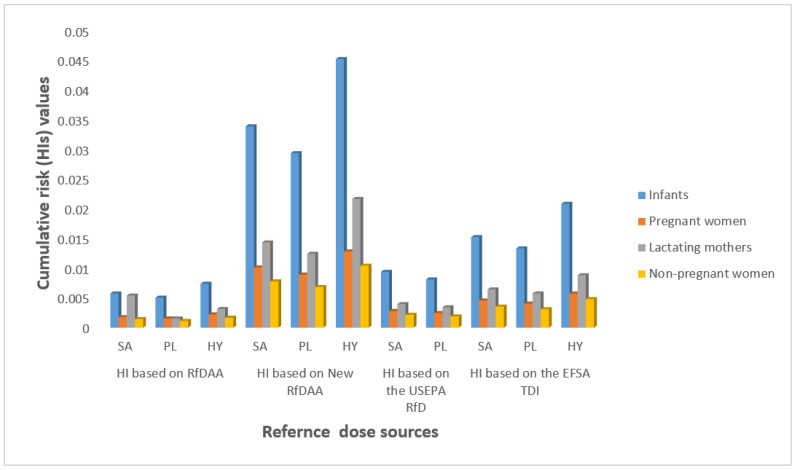
Cumulative risk of PAEs via ingestion of tap water for vulnerable groups in provincial waterworks. SA: Sadao provincial waterworks, PL: Phang La provincial waterworks; Hat Yai: Provincial waterworks; HI based on RfDAA: Hazard Index of PAEs obtained by RfDAA; HI based on new RfDAA: Hazard index obtained by new RfDAA; HI based on USEPA RfD: Hazard index obtained by USEPA RfD; HI based on EFSA TDI: Hazard index obtained by ESFA TDI.

**Figure 3:**
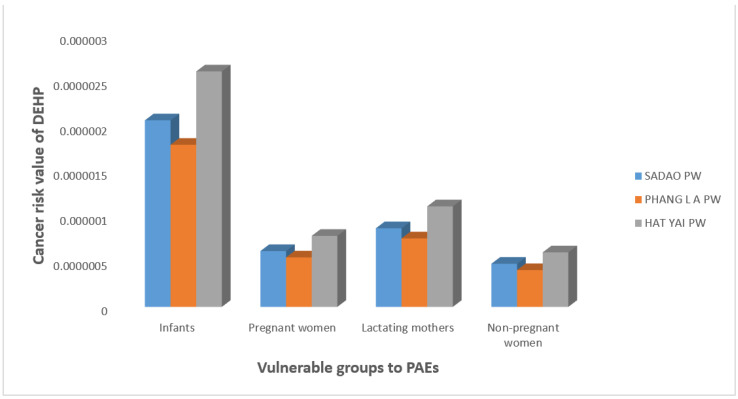
Cancer risk of DEHP via ingestion of tap water in vulnerable groups. Sadao PW: Sadao provincial waterworks, Phang La PW: Phang La provincial waterworks; Hat Yai PW: Hat Yai Provincial waterworks.

In this present work, we estimated the daily intake of PAEs by using the maximum concentration values of PAEs measured in tap water samples. These values are indicated in Table 4. We used the worst-case scenarios to estimate the EDIs, HQs, CVDs, and HIs of DBP, DEHP, and DiNP for the three sensitive groups. In addition, we estimated the ECR of DEHP. Thus, Table 4 presents exposure assessment values of target PAEs in the three different vulnerable groups, including infants, pregnant women, and lactating mothers as well as nonpregnant women. Figure 2 and Figure 3 present the cumulative risks of measured PAEs and the cancer risk of DEHP, respectively.

As shown in Table 4, the estimated chronic daily intakes (EDI) of DEHP, which is the congener with the highest MC in HY waterworks (worst scenario) were 1.86 × 10^−1^ for infants, 7.9 × 10^−2^ for lactating mothers, 5.6 × 10^−2^ for pregnant women, and 4.3 × 10^−2^ (µg/kgbw/day) for non-pregnant women. For DBP, the highest EDIs were 1.61 × 10^−1^ for infants, 6.8 × 10^−2^ for lactating mothers, 4.8 × 10^−2^ for pregnant women, and 3.7 × 10^−2^ (µg/kgbw/day) for non-pregnant women. For DiNP, the EDI highest values were 1.49 × 10^−1^ for infants, 6.3 × 10^−2^ for lactating mothers, 4.5 × 10^−2^ for pregnant women, and 3.4 × 10^−2^ (µg/kgbw/day) for non-pregnant women.

In this present study the estimated hazard quotients (HQs), as indicated in Table 4, did not exceed 0.0240, indicating that the calculated HQs for all the target groups were well below 1. In addition, this value was less than 0.2 for the single human exposure pathway of PAEs. Thus, it can be inferred that exposure to target PAEs via tap water does not pose health risks associated with antiandrogenicity because an HQ value of less than 0.2 for any given pathway is often considered acceptable, whereas HQ values >0.2 are likely to pose adverse health effects [51].

As indicated in Figure 2, the highest hazard index (HI) based on the HINRfDAA for infants came to a value of 0.0451, which was <1 in the worst-case scenario, indicating that antiandrogenic effects are not likely to occur for combined exposures to the three congeners of PAEs at the maximum exposure level and can be ignored. With a maximum HQ of 0.024, DBP contributed 52.2% of the HI, followed by DEHP (44.4%) and DiNP (3.4%). Our results show the increasing HIs that followed the order of HINRfDAA > HITDI > HIRfD > HIRfDAA. Relative to the former three reference doses (HITDI, HIRfD, and HIRfDAA), the new proposed NRfDAA yielded higher HI estimates, indicating its greater protective capacity as a tool than other RfDs, particularly the former RfDAA. In addition, the revised RfDAA indicates that both DEHP and DBP are co-drivers of HI, contrary to DEHP being the main driver of HI, as is often revealed by RfDAA. These results are in agreement with [42].

In view of the contribution of water to the daily intake of PAEs, our findings reveal that the daily intake of PAEs via drinking water did not exceed 0.37% of the TDI for DEHP in infants. In addition, the nearest concentration of an individual PAEs to the TDI was observed for DBP (1.61%), suggesting that the exposure level is very low and safe. Our result is consistent with previous findings [51,52,53].

In addition, as shown in Figure 3, the excess lifetime cancer risk triggered by exposure to DEHP via drinking tap water, particularly in infants, was higher in HY than in SA and PL waterworks. Nevertheless, in comparison to the established criterion (less than 10^−6^), the calculated excess lifetime cancer risk in infants is acceptable and can be ignored.

## 4. Discussion

PAEs are considered to be estrogenic and antiandrogenic endocrine disruptors, which can migrate from plastic pipes used in building water treatment plants into tap water. Additionally, raw waters are a major contributor of PAE contamination of tap water [10,14,16]. Moreover, poor PAE removal efficiency, particularly by conventional water treatment processes, has been observed to contribute to the presence of PAEs in tap water. Furthermore, humans can be easily exposed to PAEs via drinking contaminated tap water, posing health risks, particularly to the vulnerable population [13,15]. Thus, the aim of this study was (1) to evaluate the concentration of PAEs in tap water, (2) to assess the PAE removal efficiency of waterworks that use conventional processes, and (3) to evaluate the chronic daily intake of PAEs and cumulative health risk of PAEs in vulnerable groups via tap water intake based on four reference sources.

The variable composition of PAEs may indicate different sources. Evaluation of the individual PAE compositions is helpful in tracing pollutants’ sources and in indicating the transport and fate of PAEs in water [13]. The variation of PAE congener profiles in this study may be attributed to source compositions, solubility of water, and environmental degradation. In addition, our findings are consistent with previous studies that reported the predominance of DEHP distribution patterns in each waterwork, reflecting the different patterns of plastic contaminant input during the sampling period [13,15]. Several residential, commercial, agricultural, and industry areas are major sources of PAE pollution in water. DBP and DEHP have been identified as antiandrogenic pollutants and reported to cause reproductive abnormalities, defectiveness, and underdevelopment of epididymis, the prostate, seminal vesicle, and other organs [48,54,55]. Thus, we recommend the frequent monitoring of PAEs in surface water bodies, especially those used as sources of water and tap water in developing countries including Thailand.

It was observed that conventional water treatment plants may reduce the PAE levels and their potential adverse effects to human health [50]. However, several other studies indicated that they could remove PAEs efficiently, and the levels of PAEs in drinking water of DWTPs were even higher than those in the source water [13,15]. PAE removal efficiencies in the investigated DWTPs of the Songkhla Province are shown in Table S4. As indicated, the results reveal that the removal efficiencies of DBP, DEHP, and DiNP in DWTPs varied. DiNP was the most resistant to removal with the lowest removal efficiency of 59.9%. Overall, the concentrations of PAEs in tap water were lower than the levels in raw water samples, showing the level of PAE removal by the conventional water treatment process. However, the PAE removal efficiency was poor, ranging from 59.9 to 69.1%, indicating that the conventional drinking water treatment process cannot remove these pollutants adequately irrespective of the raw water type. This is because the traditional water treatment process focuses mainly on the removal of particles and colloids. Studies on the potential transformation of PAEs during conventional water treatment processes such as chlorination are scare. Nevertheless, a study evaluated the impact of disinfectant on the structures of DEP and DEHP, using chlorination techniques in water. The formation of a new species was observed with DEP, but DEHP was reported to be recalcitrant. The transformation product of DEP was identical to monoethyl phthalate (MEP) [56]. However, studies have demonstrated that oxidation or microbial action are the principal mechanisms for the removal of PAEs in degradation in aquatic environments, including surface waters used as raw water. In addition, microbial oxidation can biodegrade and mineralize certain species of PAEs, while some are considered recalcitrant [57,58,59]. Therefore, we recommend that the application of treatment processes comprising the combination of these fundamental methods be incorporated into traditional water treatment plants. In addition, strategies to limit source contamination, such as public awareness campaigns on the reduction in the use, recycling, and indiscriminate disposal of plastic products and boiling tap water before drinking are recommended.

The maximum concentration values of PAEs in tap water were far below the levels stipulated by USEPA (6.0 µg/L) and WHO (8 µg/L) in drinking water [3]. Nevertheless, it must be acknowledged that PAEs are endocrine-disrupting chemicals whose effects even at a low concentration may alter the function of the endocrine system. In addition, the three PAEs detected herein (DBP, DEHP, and DiNP) can pose antiandrogenic effects in humans, especially vulnerable groups [37,42,46,60]. Moreover, since PAE-contaminated tap water is constantly ingested in daily life and considering their extensive use in developing countries, it was wise to evaluate the intake of PAEs via drinking tap water, particularly in vulnerable groups including infants, lactating mothers, and pregnant women.

The chronic daily intake of PAEs via tap water in this present study was in the increasing order of infants > lactating mother > pregnant women > nonpregnant women. This may be attributed to the fact that infants’ water intake is higher than their body weight and in the case of lactating mothers, increased water demand during lactation. The EDIs of DBP, DEHP, and DiNP in all the susceptible groups in this work were higher than the values reported for adults in South Africa [61]. The oral daily intakes of DBP and DEHP obtained in this study for pregnant and lactating mothers were lower than the values reported in Iran [52]. For DEHP, the oral EDI values via drinking water in this work were lower than values reported in children and lactating mothers in Iran [53], adults in India (0.027 µg/kg/day) and adult females in Taiwan (0.115 µg/kg/day) but comparable to values reported for adults in France (0.00105 µg/kg/day). Nevertheless, the estimates were also below the overall dietary intake (DI) range of 0. 08–69.6 μg/kg/day as assessed for multiple exposure pathways [3,57]. Furthermore, in all cases, the exposure was clearly below the values set by the EFSA for TDI, the US Environmental Protection Agency (USEPA) for RfDs [62,63,64,65], and antiandrogenicity reference doses (RfDAA) and new antiandrogenicity reference doses (NRfDAA) as estimated by [41,42]. As shown in Table S4 the calculated HQs for DEHP, DBP, and DiNP in tap water by using four different reference doses including those of the EFSA for TDI and USEPA for RfD, RfDAA, and NRfDAA. Our results reveal that the noncarcinogenic and antiandrogenic health risk posed by the detected PAEs to the susceptible groups was negligible and can be ignored. The intakes of individual congeners via drinking tap water were also found to be much lower than currently published RfD benchmarks. For instance, the currently published RfD for DEHP on EPA’s IRIS database is 20 μg/kg/day, whereas the estimated value regarding the intake of DEHP in infants (considered the most susceptible group) via drinking tap water in HY is 0.02 μg/kg/day (0.1% of USEPA’s recommended RfD value). Similarly, the currently recommended RfDAA and New RfDAA are 30 and 10 µg/kg/day, respectively, with 0.07% for RfDAA and 0.2% for NRfDAA. In a previous study, the intake of DEHP in children, considered the most vulnerable group for exposure via drinking bottled water was 0.1 μg/kg/day (0.5% RfD), (0.3% RfDAA), and (1% NRfDAA), which is higher than our findings [66]. However, our findings are consistent with a previous study on multiple exposure pathways. The author observed that human exposure to PAEs (DEHP, DBP, and DEP) via drinking water a single pathway was ≤0.2% [67]. Moreover, it is worthy of note that the estimated percentages for DBP and DEHP were lower than the default values of 1 and 10% used to establish the WHO guideline. Furthermore, our results reveal that maximum exposure to PAEs does not always represent worst-case assumptions. This finding is consistent with a previous study that observed cases of higher PAE exposure not directly proportional to the maximum intake values [41].

The execution of a cumulative risk assessment based on the four different hazard indices showed a negligible noncarcinogenic risk that can be ignored for all the susceptible groups and nonpregnant women. However, the overall highest His value obtained was found in infants. In infants and children, accumulation of PAEs is more likely to occur due to a less functional glucouronidation process, and prolonged exposure to PAEs at low concentration may lead to premature development of secondary sexual characters [68]. Consequently, PAEs risk may be more likely among infants than other vulnerable groups. Moreover, previous studies have reported that exposure to PAE mixtures at a critical developmental stage can lead to the impaired development of psychomotoric skills [69] and neurological disorders, such as attention-deficit syndrome, hyperactivity, or lower intelligent quotients [70]. A recent study by Papaioannou et al. [71] estimated by utilization of multiomics analyses how exposure to PAE mixtures disturbs the urea cycle and chlorine metabolism. Thus, there is a need for frequent risk monitoring of susceptible groups by the cumulative risk assessment approach. Therefore, a future cumulative risk assessment should consider simultaneous exposure to all chemicals that have antiandrogenic effects in tap water. If this is not carried out, it is likely that we will have substantially underestimated cumulative risks from these groups of chemicals in tap water.

Several studies on human exposure and health risk of PAEs in tap water have been conducted. Most of these studies focused on exposure and risk assessment of PAEs in adults (male and female) and children [13,14,15,49,61], which observed that the daily intake of PAEs via tap water caused acceptable noncarcinogenic and carcinogenic health risk. None of these studies evaluated the exposure and cumulative health risk of PAEs in other vulnerable groups such as infants, lactating mothers, and pregnant women. A combined exposure assessment could be used to comprehend the health effects of interactions between pollutant groups and to control potential adverse effects and isolate the risk of a single class of compounds [72]. Independently, several anthropogenic compounds have been connected with similar adverse health endpoints, such as altered hormonal action in pregnant woman, lactating mothers, fetuses, and infants and altered behavioral and cognitive development in children [26,73]. Additionally, several biomonitoring studies have indicated that infants, pregnant women, and lactating mothers are exposed to multiple chemicals simultaneously [29,74]. In addition, the early stages of an infant’s life are regarded as their most vulnerable periods for exposure to PAEs [74]. In view of the fact that humans, especially vulnerable groups, can be exposed to PAEs through diverse sources, the aim of this present work was to identify if tap water is a major source of PAE intake in Songkla Province. Our findings indicate that exposure to PAEs via tap water in vulnerable groups and non-pregnant women is low and can be considered safe for now, even in tap water samples collected from HY, the waterworks with the highest MC levels of PAEs. However, the risk based on the combined exposure to three PAEs via tap water was higher than that posed by single compounds.

As shown in Figure 3, the carcinogenic risk posed by the level of DEHP via the ingestion of tap water was found to be far below the acceptable risk level (10−6) for cancer risk. For reference, the level of DEHP in tap water corresponding to an excess estimated lifetime cancer risk of 1 in 1,000,000 is 0.000026 μg/L. In fact, the carcinogenic risk posed by the highest concentration (worst-case scenarios) of DEHP in tap water is negligible in all the vulnerable groups and nonpregnant women, and can be ignored for now. Though the cancer risk of DEHP obtained in this present study was considered safe for all the susceptible populations, environmental exposures of humans, especially the vulnerable populations, to DEHP and its primary metabolites have been associated with cancer risk; a literature review of DEHP genotoxicity and potential carcinogenic mechanisms stated that this pollutant can induce cancer risk at concentrations lower than those inducing apoptosis or necrosis. These cancer risks include damage to DNA and chromosomes, amplified transformation, reverse apoptosis in tumor cell lines and then in nuclear receptors, increased cancer progression, and gene expression changes observed at low concentrations [75]. In addition, DEHP has been classified as a probable carcinogen [75], and its usage is gradually being reduced in developed countries due to this reason.

### Limitations and Strengths

This study has a number of significant limitations and strengths. Clearly, differences in RfDs may arise due to different methods used in assigning these values, such as the application of default uncertainty factor values; availability of data including physiologically based pharmacokinetic models; and the size of the literature database. Derivation of RfDs incorporates the consideration of uncertainty; certainly, the USEPA description of the RfD as “an estimate that have uncertainty spanning probably an order of magnitude of a daily exposure to the human population including vulnerable subgroups that is likely to be without an appreciable risk of deleterious effects during a lifetime” is wisely phrased to caution against overinterpretation.

Typically, a major area of uncertainty when using the HI is the assumption of dose additivity. However, as compared to most groups of pollutants, this is less of a concern for PAEs because there are some toxicology studies which provide empirical evidence of the joint effect of cumulative exposure to PAEs in relation to adverse health effects [73]. Each of these studies reported that health effects were best predicted by a dose-additive model, giving backing to the use of the HI.

While the HQ and HI have the benefit of being easily estimated and interpreted, there are also uncertainties associated with their use. Firstly, they provide only a single number to describe health risk, because of the usage of a single reference value. Defining the distribution of health risk within a population including sensitive subgroups is a complex process that should consider variability within and between groups and the possible impact of factors including age and gender. Correspondingly, with regard to the reference values, the selection of endpoints has an obvious effect on the outcome of the exercise. The choice of using different endpoint domains for each exposure of interest is based upon the quality of the database as well as the magnitude of the reference value. Clearly the value of the RfDs can vary widely depending on such factors. As revealed by this present study, DEHP exposure dominates the HI results; the results differ widely whether using the USEPA RfD or the new antiandrogenicity reference doses (NRfDAA) as estimated by [42].

We could not analyze tap water samples from other waterworks that obtain their raw water from other surface water in Southern Thailand, and the relatively small sample size precluded complex analyses or exploring interactions. In addition, the seasonal variations of PAEs were not evaluated and reported in this present study. Moreover, other forms of antiandrogenic and endocrine-disrupting chemicals (EDCs) were not analyzed in this work. Furthermore, this present work focused mainly on exposure via tap water; thus, the cumulative exposure assessment may be underestimated due to exclusion of other sources of exposure including food, cosmetics, and dust. Therefore, caution is desirable in interpreting the findings reported in this present work. Despite these limitations, to the best of our knowledge this the first study to evaluate the cumulative health risk of PAEs in susceptible groups via the ingestion of tap water, in addition, we found associations, some of which were plausible and consistent with previous studies.

## 5. Conclusions

Tap water is a major route of human exposure to antiandrogenic and endocrine-disrupting chemicals. We evaluated the concentrations and fate of PAEs in traditional drinking water treatment plants. In addition, the potential cumulative risk assessment of PAEs in sensitive subgroups in tap water were evaluated by using the EFSA’s recommended value of TDI, the US Environmental Protection Agency (USEPARfD), and antiandrogenicity reference doses (RfDAA) and new RfDAA (NRfDAA) as estimated by [41,42]. The results reveal that the maximum concentration of DEHP (4.48 µg/L) in raw water samples was higher than standard values stipulated by USEPA (PAEs, 3.0 µg/L) and WHO (DEHP, 1.3 µg/L) for surface water quality values. Additionally, the DBP maximum value of 3.36 µg/L was slightly higher than the Chinese standard limit of 3.0 µg/L for surface water. In tap water DEHP and DBP were below the drinking water standards limits. Our findings confirm the inadequate removal of PAEs by the conventional treatment process. In addition, the study revealed that the level of PAEs (DEHP, DBP and DiNP) in drinking tap water is safe and both individual and cumulative effects do not pose risks to vulnerable groups such as infants, lactating mothers, and pregnant and nonpregnant women. However, human beings can be exposed to several PAE congeners simultaneously. Thus, the risk caused by cumulative effects of exposure to several PAEs needs to be considered. Therefore, understanding exposures to mixtures across the life span (cumulative risk assessment) is critical for improving risk assessment and chemical safety.

## Figures and Tables

**Table 1:** Quality assurance/quality control parameters for the extraction and analysis of six PAEs.

PAEs	Linearity R^2^	Target Ions (*m*/*z*)	Retention Time (min)	Recovery (%) *n* = 3	RSD (%)	LOQ *n* = 7 μg/L	LOD *n* = 7 µg/L
DBP	0.999	223, 205, 167	7.57	84	5.9	0.11	0.07
BBP	0.999	205, 149, 91	8.77	69	6.2	0.07	0.03
DEHP	0.999	279, 167, 149	9.29	99	7.2	0.16	0.10
DnOP	0.999	279, 261, 149	9.84	93	6.8	0.18	0.13
DiNP	0.999	293, 127	9.93	110	7.6	0.29	0.18
DIDP	0.999	307, 141	10.44	119	8.4	0.82	0.12

PAEs: Phthalate esters; di-n-butyl phthalate (DBP), benzyl butyl phthalate (BBP), di-2-ethyl hexyl phthalate (DEHP), di-n-octyl Phthalate (DnOP), di-isononyl phthalate (DiNP), and diisodecyl phthalate (DIDP) LOD: limit of detection; LOQ: limit of quantification; RSD: relative standard deviation.

**Table 2:** PAEs concentrations in raw water.

PAEs	SADAO PW (µg/L)			PHANGALA PW (µg/L)			HAT YAI PW (µg/L)		
	Min	Max	Mean ± SD	Min	Max	Mean ± SD	Min	Max	Mean ± SD
DBP	ND	2.04	1.89 ± 0.15	ND	1.82	1.68 ± 0.18	ND	3.36	2.21 ± 0.58
DEHP	1.84	2.68	2.18 ± 0.36	1.69	2.14	1.93 ± 0.20	2.88	4.84	3.71 ± 0.82
DiNP	ND	1.74	1.47 ± 0.23	ND	1.68	1.63 ± 0.06	ND	2.47	2.04 ± 0.29
BBP	ND	ND	ND	ND	ND	ND	<LOD	<LOD	<LOD
DnOP	ND	ND	ND	ND	ND	ND	<LOD	<LOD	<LOD
DIDP	ND	ND	ND	ND	ND	ND	<LOD	<LOD	<LOD
∑PAEs	1.84	6.46	5.54 ± 0.74	1.69	5.64	5.27 ± 0.48	2.88	10.67	8.16 ± 1.69

PAEs; Phthalate esters, Sadao PW: Provinicial waterworks located at Sadao, PhangLa PW: Provinicial waterworks located at PhangLa, Hat Yai PW: Provinicial waterworks located at Hat Yai PW: ND: non-detectable; <LOD: less than limit of detection.

**Table 3:** Comparison of the concentrations of DBP and DEHP in this study with concentrations reported from other locations in the world (μg/L).

Location	Method of Treatment	Raw Water		Tap Water		References
		DBP	DEHP	DBP	DEHP	
Taiwan	Conventional treatment process	<MDL–0.76	<MDL–2.50	<MDL–0.84	<MDL–2.88	Guo et al., 2016
China	Conventional treatment process	0.05–4.49	0.13–6.57	0.02–1.71	0.05–2.36	Liu et al., 2013
China	Conventional treatment process	0.02–0.08	0.18–0.75	0.01–0.03	0.07–0.31	Kong et al., 2017
USA	Conventional treatment process	1.44–8.34	2.67–5.94	(mean, 2.73)	2.43–2.68	Loraine and Pettigrove, 2006
USA	Advanced process	0.05–0.06	0.12–0.17	ND	ND	Benotti et al., 2009
China	Advanced process	14.00–100	0.46–7.00	0.07–0.19	0.01–0.05	Hu et al., 2013
Taiwan	Advanced process	0.08–0.09	0.13–0.16	0.01–0.07	0.02–0.12	Yang et al., 2014
Thailand	Conventional treatment process	ND-3.36	1.69–4.84	ND-1.07	0.59–1.24	Present study

MDL: minimum detectable level, ND: nondetectable.

**Table 4:** Assessment of exposure to PAEs via ingestion of tap water in vulnerable subgroups.

	Sadao PW			Phangla PW			Hat Yai PW		
	DBP	DEHP	DiNP	DBP	DEHP	DiNP	DBP	DEHP	DiNP
**MC in Tap Water (μg/L)**	0.78	0.98	0.64	0.68	0.85	0.52	1.07	1.24	0.99
Infants									
EDI	1.17 × 10^−1^	1.47 × 10^−1^	9.6 × 10^−2^	1.02 × 10^−1^	1.28 × 10^−1^	7.8 × 10^−2^	1.61 × 10^−1^	1.86 × 10^−1^	1.49 × 10^−1^
$HQ_{RfD}$	1.2 × 10^−3^	7.35 × 10^−3^	8.35 × 10^−4^	1.02 × 10^−3^	6.4 × 10^−3^	6.78 × 10^−4^	1.61 × 10^−3^	9.3 × 10^−3^	1.30 × 10^−3^
$HQ_{RfDAA}$	7.8 × 10^−4^	4.9 × 10^−3^	6.4 × 10^−5^	6.8 × 10^−4^	4.3 × 10^−3^	5.2 × 10^−5^	1.07 × 10^−3^	6.2 × 10^−3^	9.93 × 10^−5^
$HQ_{NRfDAA}$	1.75 × 10^−2^	1.47 × 10^−2^	1.63 × 10^−3^	1.52 × 10^−2^	1.28 × 10^−2^	1.32 × 10^−3^	2.40 × 10^−2^	1.86 × 10^−2^	2.53 × 10^−3^
$HQ_{TDI}$	1.17 × 10^−2^	2.94 × 10^−3^	6.4 × 10^−4^	1.02 × 10^−2^	2.56 × 10^−3^	5.2 × 10^−4^	1.61 × 10^−2^	3.72 × 10^−3^	9.93 × 10^−4^
CVD	1.17 × 10^1^	2.94 × 10^−1^	6.4 × 10^−2^	1.02 × 10^1^	2.56 × 10^−1^	5.2 × 10^−2^	1.61 × 10^1^	3.72 × 10^−1^	9.93 × 10^−2^
Pregnant women									
EDI	3.5 × 10^−2^	4.4 × 10^−2^	2.9 × 10^−2^	3.1 × 10^−2^	3.9 × 10^−2^	2.4 × 10^−2^	4.8 × 10^−2^	5.6 × 10^−2^	4.5 × 10^=2^
$HQ_{RfD}$	3.5 × 10^−4^	2.2 × 10^−3^	2.52 × 10^−4^	3.1 × 10^−4^	2.0 × 10^−3^	2.09 × 10^−4^	4.8 × 10^−4^	2.8 × 10^−3^	3.91 × 10^−4^
$HQ_{RfDAA}$	2.3 × 10^−4^	1.5 × 10^−3^	1.93 × 10^−5^	2.1 × 10^−4^	1.3 × 10^−3^	1.6 × 10^−5^	2.9 × 10^−4^	1.9 × 10^−3^	3.0 × 10^−5^
$HQ_{NRfDAA}$	5.2 × 10^−3^	4.4 × 10^−3^	4.92 × 10^−4^	4.6 × 10^−3^	3.9 × 10^−3^	4.07 × 10^−4^	6.4 × 10^−3^	5.6 × 10^−3^	7.63 × 10^−4^
$HQ_{TDI}$	3.5 × 10^−3^	8.8 × 10^−4^	1.93 × 10^−4^	3.1 × 10^−3^	7.8 × 10^−4^	1.6 × 10^−4^	4.8 × 10^−3^	1.12 × 10^−3^	3.0 × 10^−4^
CVD	3.5 × 10^−1^	8.8 × 10^−2^	1.93 × 10^−2^	3.1 × 10^−1^	7.8 × 10^−2^	1.6 × 10^−2^	4.3 × 10^−1^	1.12 × 10^−1^	3.0 × 10^−2^
Lactating mothers									
EDI	4.9 × 10^−2^	6.2 × 10^−2^	4.1 × 10^−2^	4.3 × 10^−2^	5.4 × 10^−2^	3.3 × 10^−2^	6.8 × 10^−2^	7.9 × 10^−2^	6.3 × 10^−2^
$HQ_{RfD}$	4.9 × 10^−4^	3.1 × 10^−3^	3.57 × 10^−4^	4.3 × 10^−4^	2.7 × 10^−3^	2.87 × 10^−4^	6.8 × 10^−4^	3.95 × 10^−3^	5.48 × 10^−4^
$HQ_{RfDAA}$	3.3 × 10^−4^	2.1 × 10^−4^	2.73 × 10^−5^	2.9 × 10^−4^	1.8 × 10^−3^	2.2 × 10^−5^	4.5 × 10^−4^	2.6 × 10^−3^	4.2 × 10^−5^
$HQ_{NRfDAA}$	7.3 × 10^−3^	6.2 × 10^−3^	6.95 × 10^−4^	6.4 × 10^−3^	5.4 × 10^−3^	5.6 × 10^−4^	1.02 × 10^−2^	7.9 × 10^−3^	1.07 × 10^−4^
$HQ_{TDI}$	4.9 × 10^−3^	1.24 × 10^−3^	2.73 × 10^−4^	4.3 × 10^−3^	1.08 × 10^−3^	2.2 × 10^−4^	6.8 × 10^−3^	1.58 × 10^−3^	4.2 × 10^−4^
CVD	4.9 × 10^−1^	6.2 × 10^−1^	2.7 × 10^−2^	4.3 × 10^−1^	5.4 × 10^−1^	2.2 × 10^−2^	6.8 × 10^−1^	7.9 × 10^−1^	4.2 × 10^−2^
Non-pregnant women									
EDI	2.7 × 10^−2^	3.4 × 10^−2^	2.2 × 10^−2^	2.4 × 10^−2^	2.9 × 10^−2^	1.8 × 10^−2^	3.7 × 10^−2^	4.3 × 10^−2^	3.4 × 10^−2^
$HQ_{RfD}$	2.7 × 10^−4^	1.7 × 10^−3^	1.91 × 10^−4^	2.4 × 10^−4^	1.5 × 10^−3^	1.57 × 10^−4^	3.7 × 10^−4^	2.2 × 10^−3^	2.96 × 10^−4^
$HQ_{RfDAA}$	18 × 10^−4^	1.1 × 10^−3^	1.47 × 10^−5^	1.6 × 10^−4^	9.6 × 10^−4^	1.2 × 10^−5^	2.5 × 10^−4^	1.4 × 10^−3^	2.27 × 10^−5^
$HQ_{NRfDAA}$	4.0 × 10^−3^	3.4 × 10^3^	3.73 × 10^−4^	3.6 × 10^−3^	2.9 × 10^−3^	3.05 × 10^−4^	5.5 × 10^−3^	4.3 × 10^−3^	5.76 × 10^−4^
$HQ_{TDI}$	2.7 × 10^−3^	6.8 × 10^−4^	1.47 × 10^−4^	2.4 × 10^−3^	5.8 × 10^−4^	1.2 × 10^−4^	3.7 × 10^−3^	8.6 × 10^−4^	2.27 × 10^−4^
CVD	2.7 × 10^−1^	6.8 × 10^−2^	1.47 × 10^−2^	2.4 × 10^−1^	5.8 × 10^−2^	1.2 × 10^−2^	3.7 × 10^−1^	8.6 × 10^−2^	2.27 × 10^−2^

EDI: Daily intake via drinking tap water; $HQ_{RfD}$: HQ values obtained by USEPARfD; *HQ_RfDAA_*: HQ by RfDAA; $HQ_{NRfDAA}$:HQ by NewRfDAA; $HQ_{TDI}$: HQ by EFSATDI; TDI: tolerable daily intake values as estimated by the European Food Safety Authority (EFSA); RfD: the reference dose values as estimated by the USEPA; RfDAA: antiandrogenicity reference dose values as estimated by Kortenkamp and Faust (2010), NRfDAA: new antiandrogenicity reference doses (NRfDAA) as estimated by Kortenkamp and Koch (2020). CVD: dietary exposure contribution of PAEs via drinking tap water; SA: Sadao waterworks, PL: Phangla waterworks; HY: Hat Yai waterworks. PW: provincial waterworks. The four reference dose sources’ values are indicated in Table S1.

## Data Availability

The data presented in this study are available within the article or supplementary document.

## Supplementary Materials

The following supporting information can be downloaded at: https://www.mdpi.com/article/10.3390/ijerph19042187/s1. Figure S1: Chromatrograph of DBP in water samplestitle; Figure S2: Chromatrograph of BBP and DEHPin water samples; Figure S3: Chromatrograph of DnOP and DiNP in water samples; Figure S4: Chromatrograph of DIDP in water samples; Figure S5: PAEs composition of the raw water samples in investigated provincial waterworks; Table S1: values of reference dose (RfDs) sources; Table S2: body weight values and daily water consumption use for model input; Table S3: Spearman correlation matrix of individual PAEs concentration and total PAEs in raw water; Table S4: Removal efficiency of PAEs in conventional water treatment plants; Table S5: PAEs concentrations in tap water.
