# Supplementary material for: Phthalate Esters in Tap Water, Southern Thailand: Daily Exposure and Cumulative Health Risk in Infants, Lactating Mothers, Pregnant and Nonpregnant Women
Source: Int J Environ Res Public Health. 2022 Feb 15;19(4):2187. doi: 10.3390/ijerph19042187 (PMC8871872; doi:10.3390/ijerph19042187)
Supplement: Supplementary file 1 [file ijerph-19-02187-s001.zip › ijerph-1549962-supplementary.pdf]

Phthalate esters in tap water: daily exposure and cumulative health risk assessment in children, lactating mothers, pregnant and nonpregnant women

SUPPLIMENTARY INFORMATION

Table S1. values of reference dose (RfDs) sources.

Reference sources	DBP<br>µg/kg/bw/d	DEHP<br>µg/kg/bw/d	DiNP<br>µg/kg/bw/d
EFSA TDI	10	50	150
US EPA RfDs	100	20	115
RfDAA	150	30	1500
NEW RfD AA	6.7	10	59

Source EFSA (2005a, 2005b, 2005c); USEPA (2012) Kortenkamp and Faust (2010) and Kortenkamp and Koch (2020).

Table S2. body weight values and daily water consumption use for model input.

Group	Body weight<br>(kg)	Amount of water consumption (liter)
Infant	5	0.75
Pregnant women	66.3	3.0
Lactating mother	60	3.8
Nonpregnant woman	58	2

Source: Exposure Factors Handbook (EFH) and the Panel on Dietary Reference Intakes for Electrolytes, and Water by Institute of Medicine (US) (U.S.EPA 2011; DRI 2006).

Table S3. Spearman correlation matrix of individual PAEs concentration and total PAEs in raw water.

**Spearman correlation matrix**				
**PAEs**	***DBP***	***DEHP***	***DiNP***	***∑PAEs***
**DBP**	1			
**DEHP**	0.246	1		
**DiNP**	0.014	0.656*	1	
**∑PAEs**	0.518	0.869**	0.804**	1

*** Correlation is significant at the 0.05 level (2-tailed).**
**** Correlation is significant at the 0.01 level (2-tailed).**

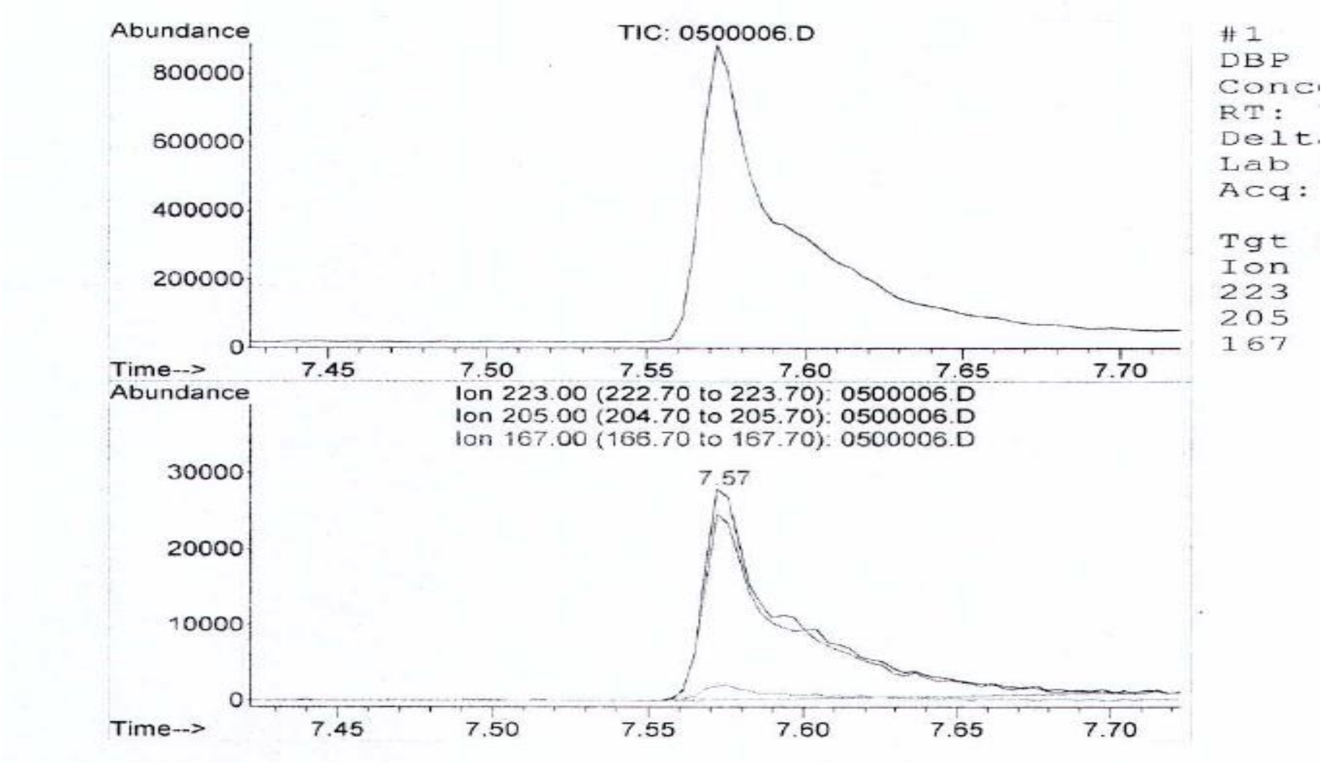

Figure S1. Chromatrograph of DBP in water samples.

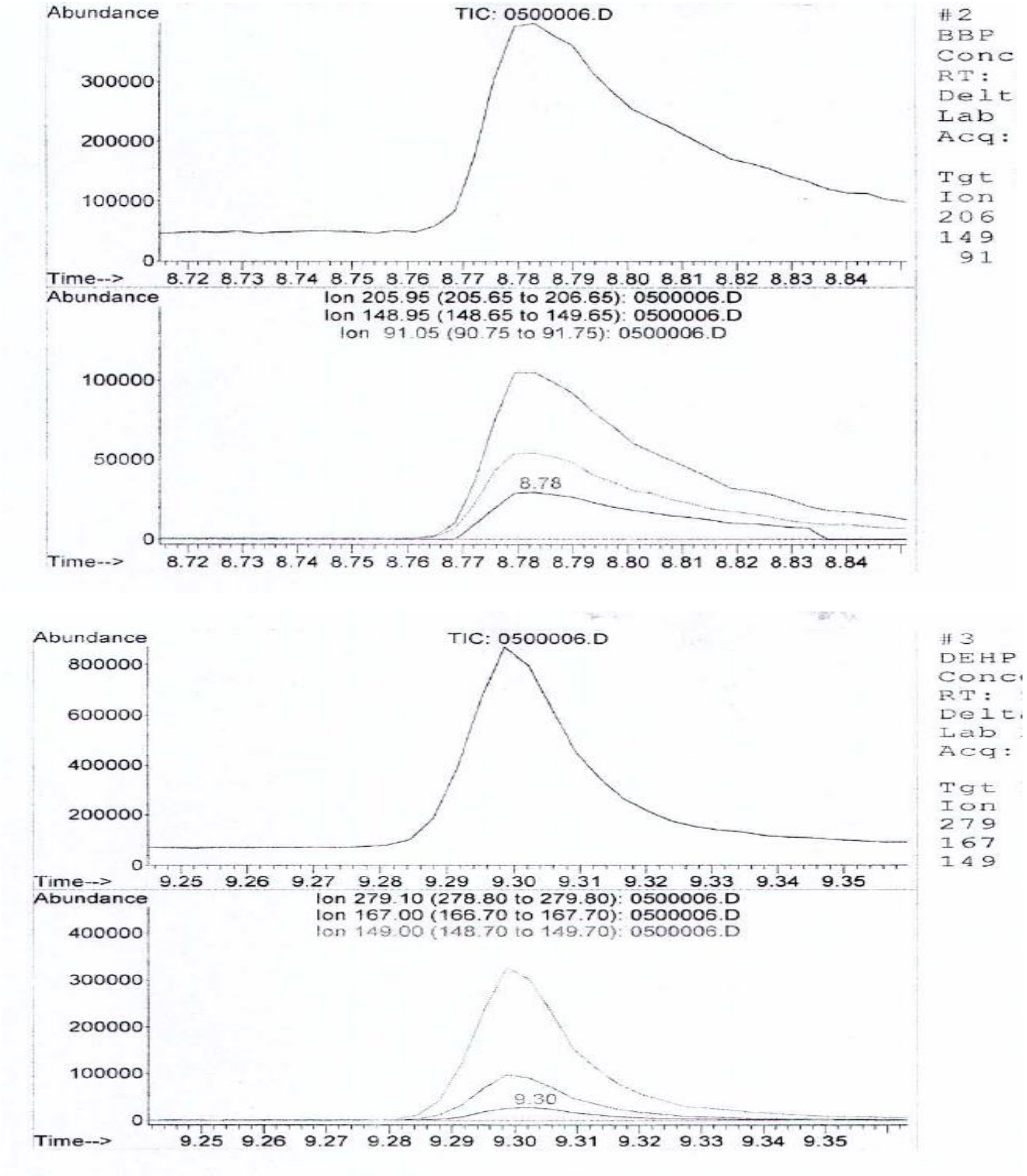

Figure S2. Chromatrograph of BBP and DEHPin water samples.

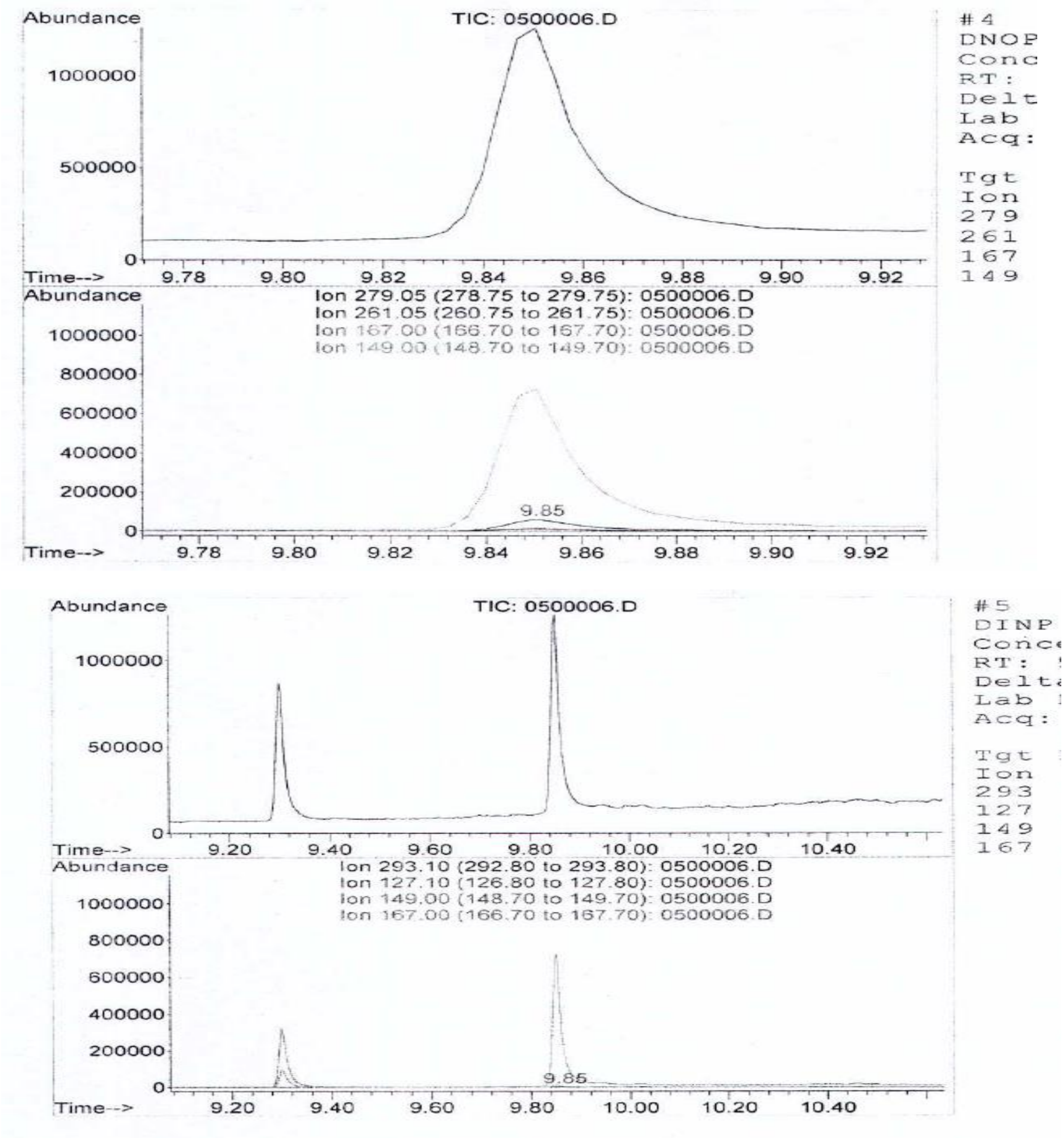

Figure S3. Chromatrograph of DnOP and DiNP in water samples.

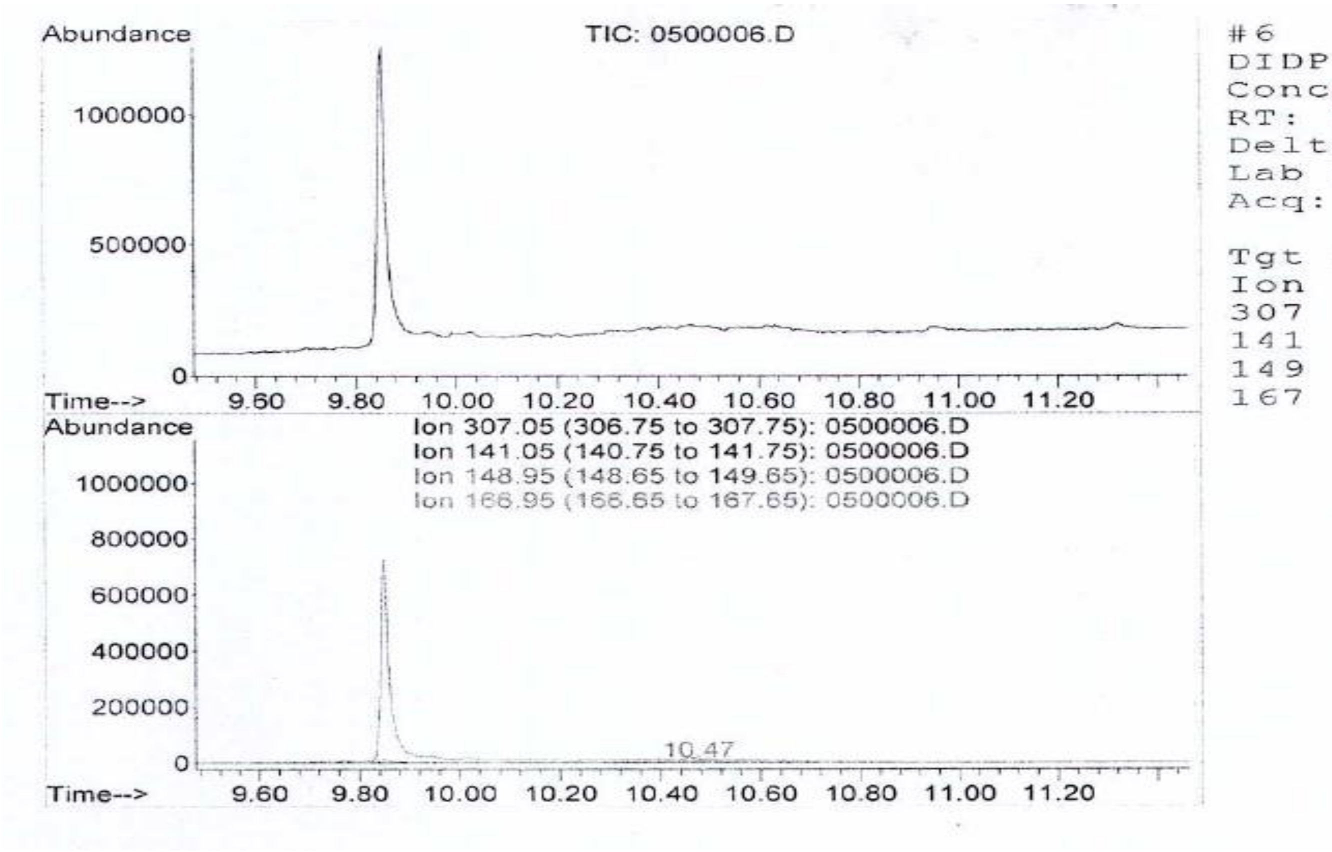

Figure S4. Chromatrograph of DIDP in water samples.

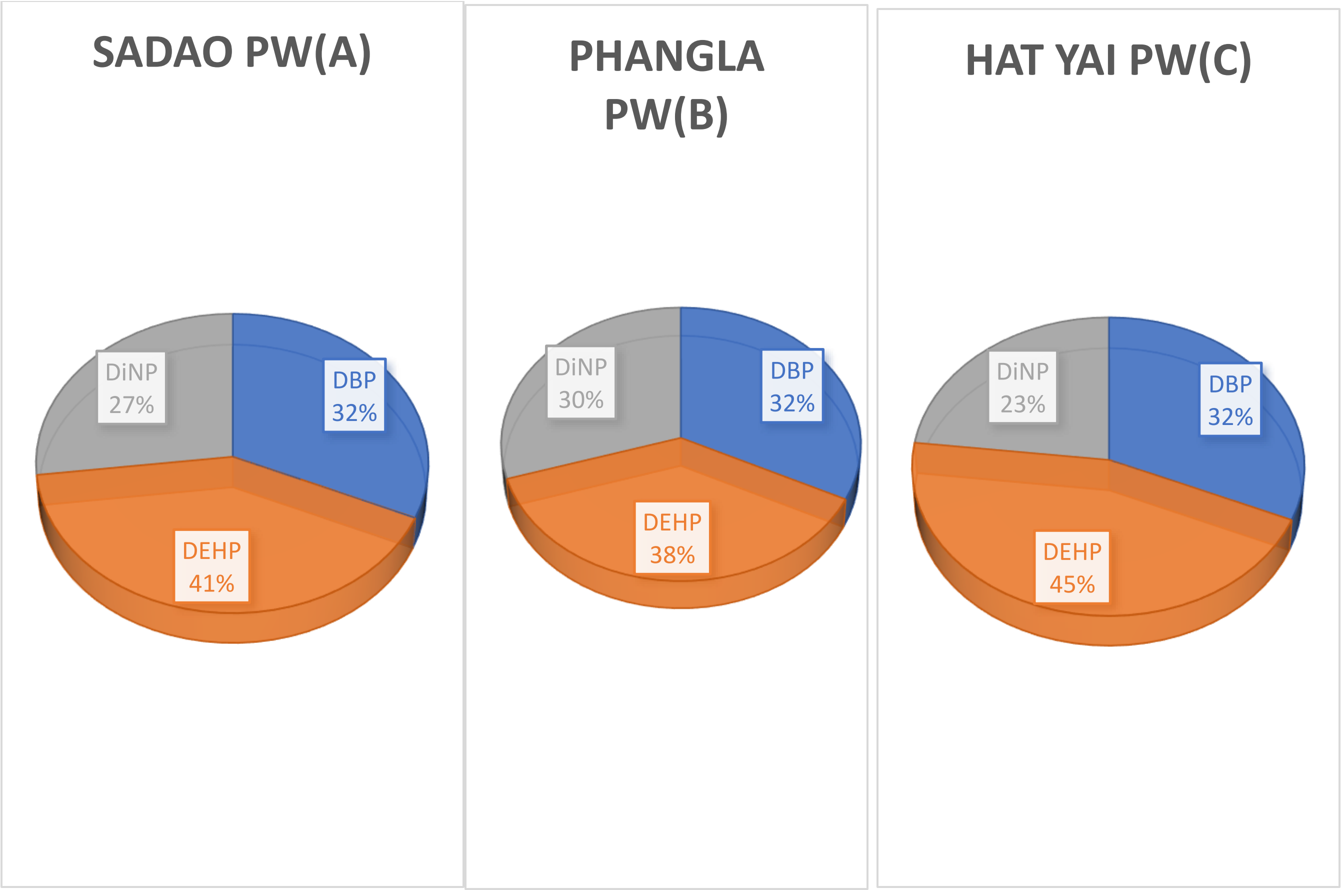

Figure S5. (**A–C**) PAEs composition of the raw water samples in investigated provincial waterworks.

Table S4. Removal efficiency of PAEs in conventional water treatment plants (μg/L).

PAEs	SADAO PW	PHANGLA PW	HAT YAI PW
DBP	1.26 (61.8%)	1.14(62.6%)	2.29(68.2%)
DEHP	1.70(63.4%)	1.29(60.3%)	3.24(66.9%)
DiNP	1.10 (63.2%)	1.16(69.1%)	1.48(59.9%)
BBP	ND	ND	ND
DnOP	ND	ND	ND
DIDP	ND	ND	ND

PW: Provincial waterworks; ND: non-detectable.

Table S5. PAEs concentrations in tap water.

PAEs	SADAO PW (µg/L)			PANGLA PW (µg/L)			HAT YAI PW (µg/L)		
	min	max	mean±SD	min	max	mean±SD	mini	max	mean±SD
DBP	ND	0.78	0.63±0.14	ND	0.68	0.54±0.12	ND	1.07	0.75±0.21
DEHP	0.59	0.98	0.81±0.17	0.53	0.85	0.69±0.16	1.02	1.24	1.12±0.09
DiNP	ND	0.64	0.58±0.08	ND	0.52	0.48±0.08	ND	0.99	0.79±0.18
BBP	ND	ND	ND	ND	ND	ND	ND	ND	ND
DnOP	ND	ND	ND	ND	ND	ND	ND	ND	ND
DIDP	ND	ND	ND	ND	ND	ND	ND	ND	ND
∑PAEs	0.59	2.40	1.98±0.39	0.53	2.05	1.71±0.36	1.02	3.30	2.62±0.48

PW: Provincial waterworks; ND: non-detectable.

References

DRI. Dietary reference intakes: the essential guide to nutrient requirements DRI, dietary reference intakes for water, potassium, sodium, chloride, and sulfate. Institute of Medicine of National Academy Press 2006.

EFSA. Opinion of the Scientific Panel on food additives, flavourings, processing aids and materials in contact with food (AFC) related to Di-isononylphthalate (DINP) for use in food contact materials. DOI:https://doi.org/10.2903/j.efsa.2005a.244.

EFSA. European Food Safety Authority, Opinion of the scientific panel on food additives, flavourings, processing aids and materials in contact with food (AFC) related to dibutylphthalate (DBP) for use in food contact materials. doi:10.2903/j.efsa.2005b.242.

EFSA. Opinion of the scientific panel on food additives, flavorings, processing aids and materials in contact with food (AFC) related to bis (2-ethylhexyl) phthalate (DEHP) for use in food contact materials. European Food Safety Authority journal. 2005c doi:10.2903/j.efsa.2005.243.

Kortenkamp,A.,& Faust, M. Combined exposures to antiandrogenic chemicals: steps towards cumulative risk assessment. International Journal of Andrology. 2010, 33(2), 463–474.

Kortenkamp, A. and Koch H.M. Refined reference doses and new procedures for phthalate mixture risk assess-ment focused on male development toxicity. Int. J. Hyg Environ. Health. 2020, 224 113428. doi.org/10.1016/j.ijheh.2019.113428.

U.S.EPA. Exposure Factors Handbook 2011 Edition (Final). U.S. Environmental Protection Agency, Washington, DC, EPA/600/R-09/052F. 2011.

U.S.EPA. Integrated Risk Information System (IRIS), U.S. Environmental Protection Agency. Di (2-ethylhexyl) phthalate (DEHP) (CASRN 117–81-7). 2012.
